# Minicollagen cysteine-rich domains encode distinct modes of polymerization to form stable nematocyst capsules

**DOI:** 10.1038/srep25709

**Published:** 2016-05-11

**Authors:** Anja Tursch, Davide Mercadante, Jutta Tennigkeit, Frauke Gräter, Suat Özbek

**Affiliations:** 1 University of Heidelberg, Centre for Organismal Studies, Department of Molecular Evolution and Genomics, Im Neuenheimer Feld 329, 69120 Heidelberg, Germany; 2 Heidelberg Institute for Theoretical Studies, Schloss-Wolfsbrunnenweg 35, 69118 Heidelberg, Germany; 3 University of Heidelberg, Interdisciplinary Center for Scientific Computing, INF 368, 69118 Heidelberg, Germany

## Abstract

The stinging capsules of cnidarians, nematocysts, function as harpoon-like organelles with unusual biomechanical properties. The nanosecond discharge of the nematocyst requires a dense protein network of the capsule structure withstanding an internal pressure of up to 150 bar. Main components of the capsule are short collagens, so-called minicollagens, that form extended polymers by disulfide reshuffling of their cysteine-rich domains (CRDs). Although CRDs have identical cysteine patterns, they exhibit different structures and disulfide connectivity at minicollagen N and C-termini. We show that the structurally divergent CRDs have different cross-linking potentials *in vitro* and *in vivo*. While the C-CRD can participate in several simultaneous intermolecular disulfides and functions as a cystine knot after minicollagen synthesis, the N-CRD is monovalent. Our combined experimental and computational analyses reveal the cysteines in the C-CRD fold to exhibit a higher structural propensity for disulfide bonding and a faster kinetics of polymerization. During nematocyst maturation, the highly reactive C-CRD is instrumental in efficient cross-linking of minicollagens to form pressure resistant capsules. The higher ratio of C-CRD folding types evidenced in the medusozoan lineage might have fostered the evolution of novel, predatory nematocyst types in cnidarians with a free-swimming medusa stage.

Collagens are a major class of extracellular matrix proteins with extended regions of Glycine-X-Y repeats, with X being mostly occupied by proline and Y by 4-hydroxyproline. They are a hallmark of the animal connective tissue and their evolution is tightly coupled to multicellularity^1^,^2^. The folding of the collagen triple helix is controlled by non-collagenous (NC) trimerization domains adjacent to the collagen sequence that are also responsible for chain selection in heterotrimeric collagens^3^. Cnidarian mesoglea collagens possess extended triple helix sequences and conserved C-terminal NC1 domains and therefore share general features with vertebrate extracellular matrix (ECM) collagens^4^. The cnidarian minicollagens stand out due to their extreme shortness and restriction to the structure of the stinging organelles, the nematocysts. How their evolution is linked to ECM collagens is a matter of debate.

Nematocysts or cnidocysts are complex organelles uniquely found in the phylum Cnidaria^5^. They consist of round or cylindrical capsules extending at one end into a long tubule structure, which is coiled inside the capsule matrix. Triggering of nematocyst discharge by chemical and mechanosensory stimuli induces tubule evagination and toxin release in an ultrafast, harpoon-like fashion offering an efficient mechanism for predation^6^,^7^. The driving force of this explosive secretory process is the high osmotic pressure of about 150 bar inside the capsule^8^. Consequently, an unusually stable protein network is required to withstand the involved physical forces.

As reported previously, the morphological complexity of nematocyst types, which is higher in medusozoans than in anthozoans, is related to their molecular complexity^9^. The protein composition of *Hydra* nematocysts has been analyzed by mass spectrometry and revealed a significant number of extracellular matrix-like proteins^10^. Minicollagens constitute a prominent fraction of the capsule wall and tubule proteome and have been subject of detailed structure-function analyses^9^,^11^,^12^. Minicollagens comprise a short central collagen sequence (12–16 Gly-X-Y repeats) flanked by variable polyproline stretches and N- and C-terminal CRDs (Fig. 1A). The CRDs have a conserved pattern of 6 cysteines (CX_3_CX_3_CX_3_CX_3_CC), which have been proposed to function in the formation of intermolecular disulfide bridges between minicollagen monomers during nematocyst maturation^13^,^14^,^15^.

Although minicollagen N-CRDs and C-CRDs have identical cysteine patterns, NMR analysis has revealed that they exhibit dramatically different folds and intramolecular disulfide connectivity signified by a *cis* to *trans* conversion of a conserved proline at position 11 (Fig. 1A,B)^16^,^17^. The two structures are connected in sequence space by single mutations favoring either the N-CRD or C-CRD folding pathway^18^. Substitutions in the N-CRD sequence at position 4 (to Val/Ile) and 14 (to Pro) are sufficient to switch the respective N-CRD fold to the C-CRD fold^18^ (Fig. 1A,B).

The functional role of the different CRDs at N and C-termini of minicollagens for collagen folding and capsule maturation has hitherto remained elusive. Here we show that the C-CRD, similar to cystine knots in fibril-associated collagens with interrupted triple helices (FACITs), participates in at least two simultaneous intermolecular links, thereby acting as a trimerization domain in the secretory pathway. This diverged function of the C-CRD is directly related to the higher accessibility of its disulfides compared to those in N-CRDs that are monovalent. Additionally, the Cys9-Cys18 C-CRD disulfide bond may possess a catalytic function influencing the allover polymerization kinetics during capsule maturation. The resulting increase in polymerization kinetics and polymer density caused by the C-CRD apparently contributed to the striking resistance of nematocysts in cnidarians, preferably in the medusozoan lineage characterized by highly pressurized capsule types. Interestingly, the expansion of the minicollagen family in medusozoans is accompanied by an increase of C-terminally located C-CRD sequence types, which probably served as a driving force for the evolution of novel nematocyst types in this clade.

## Results

### The C-CRD functions as a collagen cystine knot

**Figure 1:**
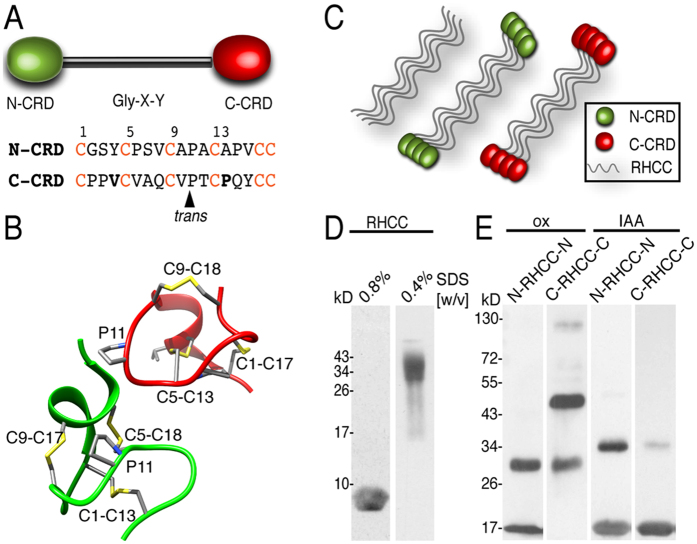
Disulfide-linked oligomers formed by N-CRD and C-CRD fusion proteins with the tetrameric RHCC. (**A**) Schematic representation of minicollagen-1 domain structure. The N-CRD is shown as green and the C-CRD as a red oval, the central collagen domain as a black bar. Lower panel: N-CRD and C-CRD amino acid sequences. The conserved cysteines are highlighted in red. Amino acids favoring the C-CRD fold are shown in bold. (**B**) N-CRD (green) and C-CRD (red) structures, secondary structure is shown in ribbons, disulfide bonds and the P10 and V4 residues responsible for the C-CRD fold are shown as sticks and colored by atom types (carbon, sulfur and nitrogen atoms are shown in grey, yellow and blue respectively). (**C**) Schematic representation of the C-CRD and N-CRD RHCC fusion proteins. (**D**) Tetrameric coiled-coil formation by the RHCC protein can be visualized in SDS-PAGE using low SDS concentration (right lane, apparent MW of about 35 kDa). Monomeric RHCC (left lane) has a calculated MW of 7,6 kDa and an apparent MW of about 9 kDa. (**E**) Disulfide-linked oligomers of RHCC-CRD fusion proteins in non-reducing SDS-PAGE (left lanes). IAA treatment blocks cysteine cross-linking (right lanes). Note that some residual dimers are present indicating incomplete modification with IAA.

CRD peptides do not show any tendency for specific oligomer formation as deduced from previous NMR studies^17^,^18^,^19^. Their reoxidation at high concentrations usually leads to the formation of large aggregates. To induce directed cysteine cross-links as probably realized in minicollagens we have fused N- and C-CRD domains to both termini of the tetrameric right-handed coiled-coil domain (RHCC) from *Staphylothermus marinus*^20^ (Fig. 1C). We chose the RHCC motif as it lacks cysteines and the tetramer is stabilized exclusively by non-covalent coiled-coil interactions. By joining the CRDs in a close parallel arrangement in the RHCC tetramer, we expected to observe cysteine disulfide cross-links formed by the interaction of the CRDs. We used the NOWA CRD sequence as N-CRD motif and its G4V;K14P double mutant version as C-CRD motif^18^ for these experiments in order exclude effects from sequence differences present in N and C-CRDs from native minicollagens.

As demonstrated in Fig. 1D, the RHCC lacking CRDs (MW: 8 kDa) shows a tetramer band about 35 kDa in SDS-PAGE using a low SDS concentration to preserve the tetrameric coiled-coil. The monomeric CRD-RHCC fusion proteins have a calculated molecular mass of 14 kDa, the apparent molecular mass being slightly higher (16 kDa). When purified samples were analyzed by SDS-PAGE, the N-CRD-RHCC fusion protein showed distinct monomer and dimer bands (Fig. 1E). Tetramer bands induced by the RHCC moiety were not present as the samples were heat-denatured. Interestingly, the oxidized C-CRD-RHCC fusion protein showed a dominant trimer band (about 50 kDa) and a less pronounced dimer band but no monomeric fraction. When the respective protein samples were treated with iodoacetamide to block disulfide formation they showed mainly monomeric bands with some dimer formation indicative of residual free cysteines (Fig. 1E). These results suggest that N- and C-CRD motifs have different oligomer formation capacities. While the N-CRD was able to form intermolecular interactions resulting in disulfide-linked dimers, cross-linking by the C-CRD resulted preferably in trimers. Since this altered capacity exclusively depended on the different folds of the respective CRDs, we conclude that trimer formation by the C-CRD fold might represent a general feature.

### Structure-based analysis explains the different polymerization functions of N- and C-CRDs

To assess the structural and dynamic differences between N- and C-CRDs, we first performed molecular dynamics (MD) simulations of the partially reduced states of the two domains, starting from their NMR solution structures^16^,^18^. While the N-CRD shows a higher tendency to remain in a compact, native state close to the experimental conformation, the C-CRD reveals higher dynamics visible in the higher values of root mean square deviation (RMSD) and radius of gyration (R_G_) (Fig. S1A,B), consistent with previous observations^21^. Interestingly, reduction of individual cysteine residues along the sequence has diverse consequences on the conformational dynamics of the proteins, with the strongest destabilization of both the N- and C-CRD folds observed for the disulfide bond at the C-terminus of the respective CRD sequence (Fig. S1C,D).

**Figure 2:**
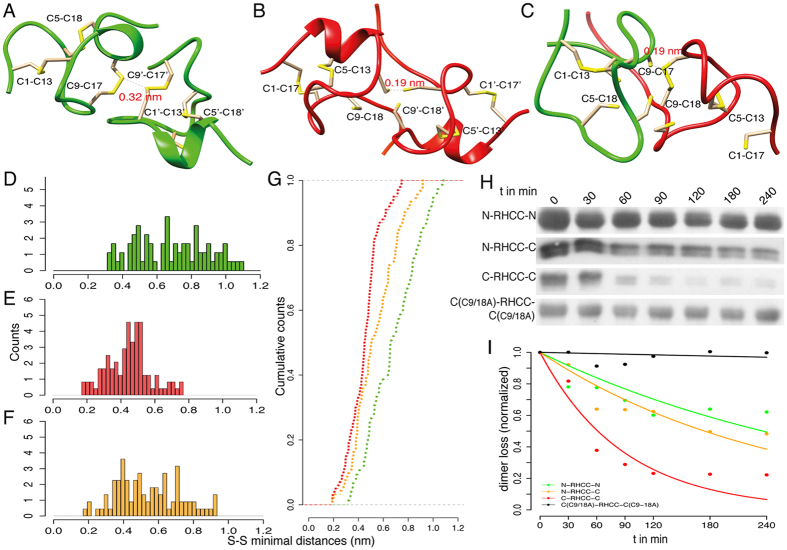
Propensity of intermolecular disulfide formation revealed by molecular docking of N-CRD and C-CRD domains. (**A**–**C**) Representative conformations of docked CRD dimers. The figures illustrate the conformations showing the lowest intermolecular S-S distance after homophilic (**A**,**B**) and heterophilic (**C**) association obtained through the molecular docking of N-CRD (**A**) and C-CRD (**B**) domains. (**D**–**F**) Distributions of the S-S minimal distances retrieved from the docking of N-CRD with N-CRD (**D**), C-CRD with C-CRD (**E**) and N-CRD with C-CRD (**F**) domains. In (**G**) the cumulative distributions relative to the histograms shown in (**D**–**F**) are reported. (**I**) The graph shows the decay of dimer as a function of time quantified from the graphical post-processing of the western blot image shown in (**H**). The sequestration of the dimer in solution is shown for the N-CRD (green) and C-CRD (red) homopolymerisation, for the N-CRD and C-CRD heteropolymerisation (orange) and for the C-CRD (C9/18A) mutant homopolymerisation (black). Solid lines show the fitting of each dataset performed using a single exponential decay model (y = e^−x/τ^).

To elucidate the molecular basis for the association preferences of the two domains observed experimentally, docking of N-CRDs and C-CRDs into homo- and heterodimers was performed. We used a flexible docking algorithm, which allowed conformational adaptations of side chains including cysteines upon complex formation. We then assessed the ability of each monomer to form inter-domain disulfides by measuring the minimal S-S distance between the docked CRDs. We obtained a large set of putative relative poses for N-N, C-C, and N-C complexes (Fig. 2). Both, N-CRD and C-CRD, were able to dock and form homodimers, in which the minimal interdomain S-S distance is compatible (<0.4 nm) with intermolecular reshuffling of S-S bonds (Fig. 2A,B,D,E). However, the C-CRD showed a more pronounced tendency to associate, as reflected by lower S-S intermolecular distances (Fig. 2B,E). The heterodimer formation showed an intermediate profile with a distribution of S-S minimal distances that includes the values encountered in the N-CRD and C-CRD homophilic docking (Fig. 2C,F). These differences are particularly evident in the cumulative S-S distance distributions (Fig. 2G).

Within the set of putative CRD-CRD homo- and heterodimers, we next identified particularly reactive cysteine residues, which are those most commonly observed to form short intermolecular S-S distances. Cysteine residues involved in the C1-C17 and C9-C18 bridges of the C-CRD exhibited a significantly higher tendency to form intermolecular disulfide bonds than others (Fig. S2), explaining the higher tendency of the C-CRD to engage in trimers or higher oligomers.

The divergent CRD functions were also investigated by monitoring the count of CRD dimers at different minimal S-S distances. While both CRDs reported the presence of three reactive patches to which a second monomer can bind, the C-CRD showed a more pronounced tendency to populate all of these patches with structures showing short S-S distances, again supporting the notion of C-CRD as a multivalent cross-linker (Fig. S3). On the contrary, for S-S distances below 3 Å, the N-CRD shows vanishing populations of poses for these patches.

The functional diversification of the C-CRD also affected the polymerization kinetics. We monitored differences in the kinetics of polymer formation by analyzing the conversion of the dimer band to polymers in different CRD-RHCC fusion proteins (Fig. 2H). Disulfide reshuffling in these cases was not initiated by adding reducing agents as this strongly accelerates the process. Instead, spontaneous cross-linking of the folded domains was monitored over time. We chose the dimer band for quantification, as monomeric C-CRD-RHCC protein is only detectable in reduced samples (see above). Quantitative dimer incorporation into the polymer was expressed as a function of time that follows an exponential decay (Fig. 2I). The sequestration of dimeric CRD-RHCC is directly related to the formation of higher-order polymers (trimers, tetramers and polymers) and therefore following the disappearance of the band relative to the dimer is an index of the appearance of longer CRD polymers. Interestingly, the polymerization trends correlate well with the differential abilities of N-CRDs and C-CRDs to form dimers as revealed by the computational docking of CRD monomers (compare Fig. 2D–F with Fig. 2I). The N-CRD dimers are transformed (τ ~ 340 min) one order of magnitude slower than the C-CRD dimers in solution (τ ~ 88 min). On the other hand, the heterophilic association of N-CRD and C-CRD leads to an intermediate rate (τ ~ 251 min) as also evidenced by the docking of N-CRD with C-CRD monomers. More interestingly, the dimer of the C9A/C18A C-CRD mutant that was specifically designed to avoid the formation of polymeric species shows a slow-down of more than one order of magnitude compared to the decay of the N-CRD homophilic dimer. Indeed, this mutant yields a dimer sequestration in the timescale of days rather than hours. Overall, these experimental results are in line with the qualitative estimation of the CRDs’ tendency to polymerize as suggested by the computational methods employed in this study.

### CRDs are promiscuous, functionally diverse polymerization domains

**Figure 3:**
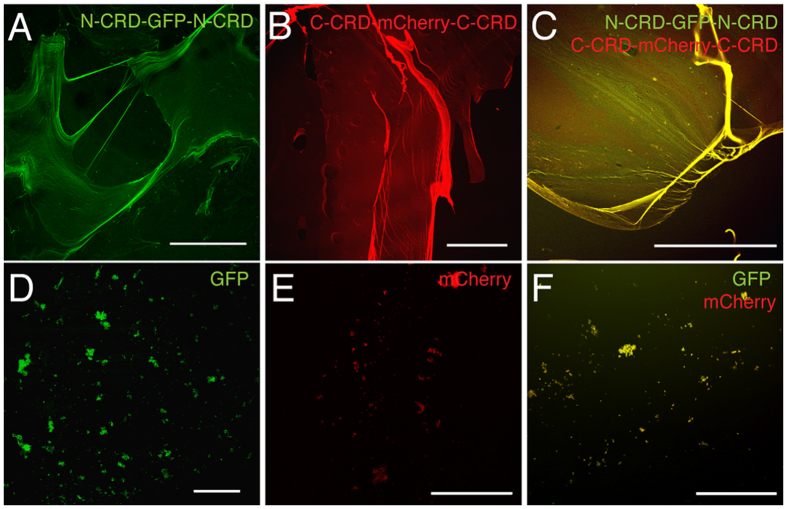
Mixed polymers formed by fluorescent minicollagen N-CRD and C-CRD fusion proteins. (**A**) Fibrous polymers formed by reoxidized GFP fusion protein carrying terminal N-CRDs. (**B**) Polymer formation of reoxidized mCherry fusion protein carrying terminal C-CRDs. (**C**) Co-polymerization of the fusion proteins from A and B results in a homogenous overlap of the fluorescent signals. GFP (**D**) mCherry (**E**) alone or in combination (**F**) do not form polymers when lacking CRDs. Scale bars: 200 μm.

Our computational studies suggest CRDs to be promiscuous, by engaging into both homo- (N-N, C-C) and heterophilic (N-C) interactions. To experimentally test this, we constructed fusion proteins of GFP and mCherry carrying either N-CRDs or C-CRDs at both termini. The purified proteins were polymerized by reoxidation at 1 mg/ml either alone or using a 1:1 ratio. The obtained polymers were coated on glass cover slips and analyzed by confocal microscopy. We found that both fusion proteins readily formed extended polymers appearing as thin, sheet-like structures with prominent stress-induced fibers (Fig. 3A,B). Thus, homophilic N-CRD as well as C-CRD cross-links were realized in polymers of heterologous CRD fusion proteins. The mixed polymer formed from both fusion proteins showed homogenously merged green and red fluorescent signals suggesting that also heterophilic cross-links between CRDs occurred (Fig. 3C). Control experiments using GFP and mCherry lacking CRD domains did not result in polymer formation (Fig. 3D–F).

**Figure 4:**
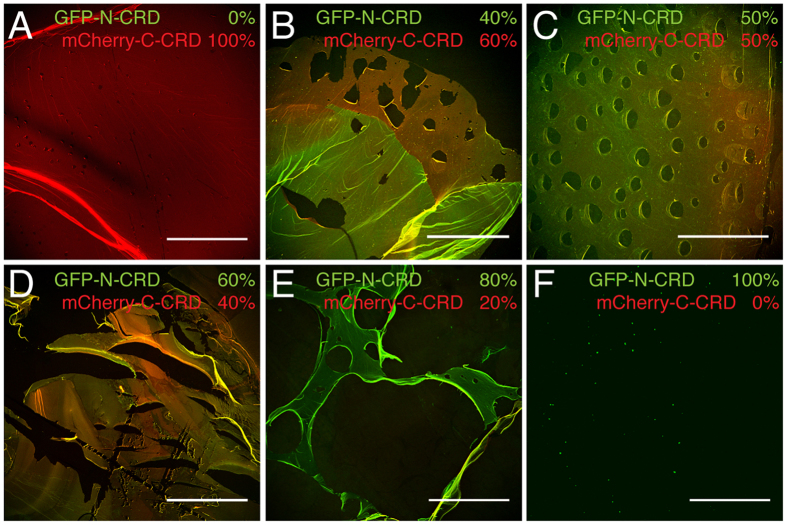
Divergent cross-linking properties of minicollagen CRDs. (**A**) An mCherry fusion protein carrying a single C-terminal CRD is still able to form extended polymers. (**B**–**E**) Polymer formation of mCherry-C-CRD is inhibited by the addition of a GFP fusion protein with a single C-terminal N-CRD in a dose-dependent manner as indicated by increased pore formation. (**F**) The GFP-N-CRD fusion protein is not able to form polymers by reoxidation indicating a restriction to single cysteine cross-links. Scale bars: 200 μm.

To exclude that the apparent heterophilic assembly mode demonstrated in Fig. 3C is an artifact, we designed GFP and mCherry fusion proteins carrying only a single C-terminal N- or C-CRD domain. Our aim was to demonstrate polymer inhibition of the N-CRD-GFP-N-CRD fusion protein by addition of the mCherry-C-CRD fusion and *vice versa*. Surprisingly, when reoxidized as a homogenous solution, the GFP-N-CRD protein did not form any polymers (Fig. 4F) while the single C-CRD fused to mCherry was still able to induce polymerization in a similar manner as the mCherry construct flanked by two C-CRDs (Fig. 4A). When we mixed the two single CRD fusion proteins at different ratios before reoxidation, we observed dose-dependent mCherry-C-CRD polymer inhibition with increasing amounts of the GFP-N-CRD protein, which could be visualized as pores of increasing number and size in the polymer texture (Fig. 4B–E). These data not only confirm that N- and C-CRDs can interact with each other and form intermolecular disulfide links, but they clearly confirm a functional diversification of the two CRDs. While the N-CRD is restricted to single intermolecular cross-links, the C-CRD is able to engage in at least two intermolecular disulfide links simultaneously.

### N- and C-CRD-specific antibodies reveal distinct assembly patterns during nematocyst morphogenesis

**Figure 5:**
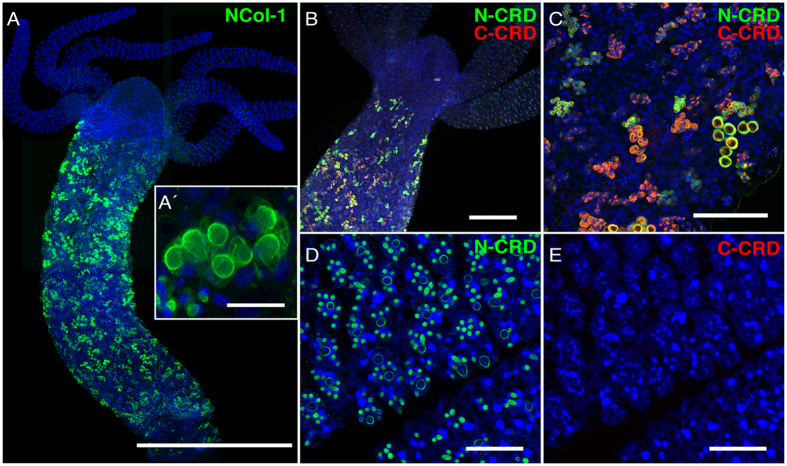
Immunocytochemistry for N-CRD and C-CRD antibodies. (**A**) Animals were stained using NCol-1 antibody (scale bar: 800 μm). (**A**) Nests of developing nematocytes are only detectable in the body column of *Hydra* (scale bar: 25 μm). (**B**) Animals were co-stained with N-CRD (green) and C-CRD (red) antibodies. Immunolocalization showed overlapping and distinct distributions of N-CRD and C-CRD signals in the body column of *Hydra*. Scale bar: 200 μm. (**C**) N-CRD and C-CRD signals in nests of differentiating nematocysts in the gastric region. Scale bar: 100 μm. (**D**,**E**) Close-up of tentacle region. N-CRD antibody stains mature nematocysts of all types in battery cells (**D**) while the C-CRD antibody does not show signals in the tentacle region (**E**). Scale bar: 50 μm. Nuclei were stained using DAPI (blue).

**Figure 6:**
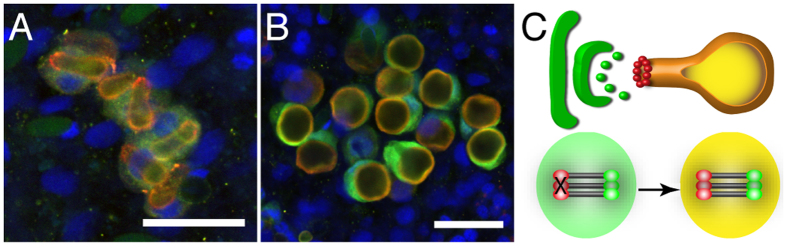
Distinct reactivities of N-CRD and C-CRD antibodies during nematocyst morphogenesis. (**A**) Close-up view of nematocyte nest in early developmental stage co-stained with both CRD antibodies. The cytoplasmic green staining indicates restricted accessibility for the N-CRD in the secretory pathway. TGN vesicles close to the apical pole of the nematocyst vesicle are marked by bright red signals of the C-CRD antibody. The capsule wall at the inner face of the vesicle membrane shows a colocalization of both signals. (**B**) Close-up view of nematocyte nest at late developmental stage. The green N-CRD staining depicts remnant NCol-1 molecules in the secretory pathway. (**C**) Schematic representation of N-CRD and C-CRD staining patterns and NCol-1 trimer formation during nematocyst morphogenesis.

Antibodies raised against CRDs in various nematocyst proteins have proven highly specific due to the low sequence similarity of the CRDs^5^,^10^,^11^,^22^. To investigate the *in vivo* assembly dynamics of the two CRDs we have raised polyclonal antibodies against N- and C-CRD peptides of *Hydra* minicollagen-1 (NCol-1) (Fig. 1A). As shown by Western blotting, the antibodies tested against the KLH-conjugated CRD peptides did not show cross-reactivity for the other CRD sequence or against KLH alone (Fig. S4). Pre-adsorption of the respective antibodies with unconjugated CRD peptides resulted in a complete loss of the signal. Immunofluorescence analysis using polyclonal anti-NCol-1 antibody raised against full-length NCol-1^23^ only stained nests of developing nematocysts in the body column of the animal (Fig. 5A). In these clusters, NCol-1 is visualized in the secretory pathway as well as in the growing nematocyst capsule (Fig. 5A′). Co-staining of *Hydra* whole mounts using both CRD antibodies revealed only partial overlap of the respective signals (Fig. 5B,C), indicating varying and discrete accessibility of the two domains during nematocyst morphogenesis. Both CRD antibodies stained clusters of developing nematocyst stages in the gastric region (Fig. 5B), but in contrast to anti-C-CRD and anti-NCol-1, anti-N-CRD also reacted with mature capsules in the tentacles (Fig. 5D,E). The notorious lack of antibody staining for mature nematocysts has been explained with capsule wall compaction by polymerization of CRD-containing proteins^11^,^13^,^14^,^22^. We therefore concluded that the N-CRD of NCol-1 is involved in disulfide-linked polymers to a lesser extend than the C-CRD. This finding was confirmed when we analyzed hydra tissue lysate and isolated nematocysts by Western blotting. Although the C-CRD antibody showed a higher reactivity for fully reduced NCol-1 in tissue lysates (Fig. S5A), minicollagen polymers from isolated capsules that were partially solubilized by DTT titration exposed more antigen binding sites at low DTT concentrations (3–6 mM) for the N-CRD antibody than for the C-CRD antibody (Fig. S5B). Nematocysts develop by an intracellular secretion process during which minicollagens and other proteins are transported via the endoplasmic reticulum (ER) and Golgi into the post-Golgi vesicle, in which the nematocyst capsule forms. The base of the capsule forms first and then the tubule forms as an extension at one end of the capsule^24^. After completion, the tubule invaginates and the capsule wall becomes compacted by polymerization of minicollagens and other CRD-containing proteins^5^. At higher magnification of morphogenetic stages visualized by both CRD antibodies, NCol-1 is detected exclusively by the N-CRD antibody in the early secretory pathway, before entering the trans Golgi network (TGN) (Fig. 6A). In contrast, the C-CRD signal emerges in vesicles of the TGN where it dominates over the N-CRD signal (Fig. 6A). After secretion into the nematocyst vesicle, the two CRD signals merge at the inner face of the vesicle membrane where the capsule wall is formed (Fig. 6B). During tubule invagination minicollagen import ceases and the secretory pathway close to the nematocyst vesicle forms a halo of residual NCol-1 (Fig. 6B). The absence of a signal for the C-CRD antibody in the early secretory pathway indicates a cross-linking of the NCol-1 molecules during this phase via their C-CRDs. Since most collagens of the extracellular matrix possess a C-terminal trimerization domain, we hypothesized that cross-linking of the C-CRDs in newly synthesized minicollagens might play a role in this process. We concluded that, different from the N-CRD, the C-CRD is engaged in intermolecular cross-links of minicollagen chains, probably acting as a C-terminal collagen trimerization motif. These initial cross-links are apparently resolved in the TGN rendering the C-CRD capable of intermolecular cross-links between different minicollagen trimers during capsule maturation (Fig. 6C).

## Discussion

The cnidarian minicollagens give rise to a specialized extracellular matrix-like supra-structure in walls and tubules of nematocysts^5^,^9^,^12^. Their assembly is controlled by a disulfide exchange reaction of their terminal CRDs at a late maturation step of nematocyst morphogenesis. This process is reversible as reducing agents lead to a dissociation of the capsule structure and release of soluble minicollagens (Fig. S5).

According to our computational analyses, all three disulfide bonds of both CRDs are solvent-exposed to an extent that they can get in proximity to cysteine residues of the bound CRD domain, in any of the possible combinations (N-N, C-C, N-C). This is in agreement with our fluorescence microscopy data (Figs 5 and 6). This is the case even for the well-structured, fully oxidized states of the CRDs, hinting towards an intermolecular S-S reshuffling mechanism that only requires very partial reduction rather than extensive reduction leading to unfolding.

Likewise, our docking studies suggest that C-CRD domains yield complexes showing lower S-S intermolecular distances, and therefore reporting for a much higher tendency to associate into polymers. This is in agreement with increased recognition of N-CRD directed antibodies in whole mounts and for isolated nematocysts (Figs 6; S5). Assuming that the accessibility of cysteine residues between structures is the primary determinant for reactivity, we find the homophilic association of C-CRD (highest reactivity) to be preferred over N-C heterodimer formation (intermediate reactivity) and even more so over N-CRD homodimer formation (lowest reactivity). Such a selective behavior cannot be reconciled with a full disruption of the native fold upon reduction, given the identical cysteine distribution along the N-CRD and C-CRD sequences. The experimental tendency of the C-CRD to function as bridging modules for the formation of cross-linked CRD polymers has been confirmed by the observation that in the C-CRD all three disulfide bridges are comparably able to be exposed and to bind a second and even a third C-CRD monomer, whereas the N-CRD fold only exposes one S-S bridge. Remarkably, the disulfide C9-C18 of the C-CRD is in a particularly ideal position to form S-S cross-links with an associated N-CRD or C-CRD domain and therefore, this disulfide bond may crucially contribute to the C-CRD’s reactivity. The high reactivity of this bond is further confirmed by our finding that the C9-C18 bond belongs to the so-called –LHStaple configuration and has a strain energy of ~33 kJ/mol according to the definition by Schmidt *et al*.^25^ (Fig. S6). Therefore, the position and the particularly strained configuration of the C9-C18 bond suggests a catalytic function, which would also explain the extremely retarded polymerization kinetics of the respective mutant protein (Fig. 2).

As in vertebrate collagens, minicollagen biosynthesis and folding is associated with the ER and the secretory pathway. In most collagens, trimerization and chain selection is initiated shortly after biosynthesis by C-terminal NC domains^3^. Hitherto it was speculative whether the minicollagen CRDs can act as trimerization motifs for the short collagen triple helix. Here, we provide evidence that the C-CRD forms intermolecular cross-links in the early secretory pathway during nematocyst morphogenesis. These interactions are apparently converted in TGN vesicles entering the nematocyst, thereby rendering the C-CRDs accessible for antibody detection. This process is possibly controlled by a nematocyst-specific disulfide isomerase^10^,^26^. Since isolated CRDs do not produce any specific multimers, minicollagen C-CRDs might have a similar function as cystine knots in FACIT collagens, e.g. collagen XII. In contrast to the C-terminal NC domains of fibrillar collagens the small NC2 domains of FACIT collagens form disulfide bonds after triple helix formation of the short interrupted collagenous domains^27^,^28^,^29^,^30^. It is therefore likely that in cnidarian minicollagens nucleation of the triple helix precedes association of the CRDs and that C-CRDs stabilize the triple-helix by forming intermolecular disulfide bonds between the three collagen chains during their secretory route to the nematocyst vesicle. The resolving of the initial cross-links stabilizing the triple helix is probably a prerequisite for the later maturation process indicating that linear cross-links are inhibited by C-CRD trimerization as a means for precluding premature polymerization in the secretory pathway. According to our immunohistochemical data, the N-CRD is not involved in a similar process. The reason might be that cross-linking at the N-terminus is blocked by the charged propeptide that precedes the N-CRD and gets cleaved closely before secretion of the collagen trimers into the nematocyst vesicle^24^. In addition, our experiments using fusion constructs of CRDs with a tetrameric coiled-coil domain indicate that the C-CRDs, when closely aligned in a parallel fashion, preferably form disulfide-linked trimers while the N-CRD forms dimers (Fig. 1C–E).

In the final capsule wall polymer, minicollagens form linear oligomers via their terminal CRDs. Kurz *et al*. initially proposed a model in which minicollagen trimers form linear fibers that are extensively cross-linked via their CRDs^31^. Our experimental data strongly support this hypothesis by demonstrating that cross-links are mainly accomplished by the C-CRD due to its ability to form at least two simultaneous disulfide bonds. Furthermore, Holstein *et al*. have postulated that fibrous minicollagens polymerized via their CRDs emanating from the base of the nematocyst form intersecting layers along the capsule body, thereby providing tensile strength in all directions^32^. This in line with our observations that CRD-coupled fluorophores form fibers in the micrometer range upon exposure to mechanical stress, indicating tear resistance over large distances.

As nematocysts evolved early in evolution, probably from extrusive organelles in protozoan precursors^10^,^33^, their biomechanical requirements might have been a driving factor in collagen evolution. Minicollagen exons, which include as few as 12 triplets, appear to be closely related to the 54 bp (6 triplets) founder exon of the collagen gene^34^. Indeed, we have recent evidence that in the starlet sea anemone *Nematostella vectensis* a large mesoglea collagen contains a minicollagen CRD (unpublished data), indicating a possible relation between nematocyst and ECM collagens.

In summary, our data reveal a unique dual function achieved by the C-CRD motif that combines the functions of collagen trimerization domain with that of a universal and highly efficient polymerization motif within a sequence of 18 amino acids. This feature not only bears important implications for biotechnological application as CRDs with different cross-linking properties can be applied as versatile polymerization conjugates for block polymers with tunable properties. It also represents an example for a significant functional gain on the organelle level by minute mutational steps during evolution. Among cnidarians, the C-CRD is more widespread in minicollagens of medusozoans than of anthozoans, where 29% have an N-CRD motif at the C-terminal position (7% in medusozoa) (Fig. S7). This indicates a stronger constraint on the mechanical resistance of the capsule structure in the medusozoan lineage. In contrast to anthozoan nematocysts, medusozoan capsule types increase their osmotic pressure to 15.3 mPa closely before discharge^35^. As diversification in minicollagens is intimately linked to the evolution of the nematocyst^9^, the demonstrated functional gain of the C-CRD, which is prevalent in the medusozoan lineage, might have been instrumental for larger evolutionary steps in the cnidarian phylum. We therefore propose that the appearance of the C-CRD motif may have promoted the invention of highly pressurized capsule types like the hydrozoan-specific stenoteles, enabling cnidarians to become efficient predators during the free-swimming medusa stage that evolved in the medusozoan clade.

## Materials and Methods

### Cloning of CRD expression constructs

For RHCC fusion constructs, the N-CRD of NOWA was amplified by PCR using the pGEV2-N-CRD construct^18^ as template (forward: TTTAGATCT ACT GGA ACA TGT CCT TCT GG, reverse: TTT GAA TTC GAT AGA AGC CAG GAT GGT AG), digested with BglII/EcoRI and ligated into the pET15b-RHCC^20^ vector. The C-terminally positioned N-CRD sequence was amplified using primers (forward: TTT GAA TTC GGT GGT GGT ACT GGA ACA TGT CCT TCT GG, reverse: TTT GAA TTC TTA TAA ATT GAC CTG ACC ACA AC) that introduce an N-terminal GGG-linker and a C-terminal stop codon. The EcoRI digested fragment was ligated into the pET15-N-CRD-RHCC construct linearized with EcoRI yielding the final pET15-N-CRD-RHCC-N-CRD expression vector.

To obtain C-CRD-containing RHCC fusion constructs, the N-CRD fold was converted into the C-CRD fold by two amino acid changes^18^. For this purpose, site-directed mutagenesis was performed on pET-RHCC-N-CRD to introduce GGT→GTT and AAA→CCA mutations, respectively. The C-CRD was amplified by PCR (forward: TTTGAATTC ACT GGA ACA TGT CCT TCT GTT TGC AGT GG; reverse: CGC GAA TTC TTA TAA ATT GAC CTG ACC ACA ACA TCC) containing EcoRI sites and was cloned into the pET15-RHCC-C-CRD vector. All constructs were confirmed by automated sequencing.

For GFP fusion constructs, the pET15b-GFP vector was mutagenized to delete the stop codon thereby introducing an NdeI site. The N-terminal N-CRD fragment containing NdeI sites was amplified by PCR using the described pET15-N-CRD-RHCC construct as template and cloned into the mutagenized pET15b-GFP vector. The C-terminal N-CRD fragment was amplified using primers that introduce terminal EcoRI sites and a 3′ stop codon (forward: TTT CAT ATG ACT GGA ACA TGT CCT TCT GGT TGC AGT GG; reverse: CGC CAT ATG TAA ATT GAC CTG ACC ACA ACA TCC) and cloned into the pET15b-N-CRD-GFP vector. For mCherry fusion constructs a similar strategy was applied using following primers for the N- and C-terminal C-CRDs: (N-terminal C-CRD coding fragment with terminal NdeI sites: forward: TTT CAT ATG ACT GGA ACA TGT CCT TCT GTT TGC AGT GG; reverse: CGC CAT ATG TAA ATT GAC CTG ACC ACA ACA TCC; C-terminal C-CRD coding fragment with terminal BamHI sites: forward: TTT GGA TCC ACT GGA ACA TGT CCT TCT GTT TGC AGT GG; reverse: CGC GGA TCC TTA TAA ATT GAC CTG ACC ACA ACA TCC).

### Protein expression, purification, and polymer formation

*E. coli* BL21(DE3) cells were transformed with pET15b expression plasmid (Addgene) coding for N-CRD-GFP-N-CRD, C-CRD-mCherry-C-CRD, GFP or mCherry. Liquid cultures were induced with IPTG to a final concentration of 1mM at an OD600 = 0.5 and incubated for further 3,5 hours. Bacteria were harvested by centrifugation (6000 × g, 15 min, 4 °C). The pellet was resuspended in 1 × PBS containing protease inhibitor mix (Roche), sonicated and subsequently centrifuged at 22000 × g, 30 min, 4 °C. The supernatant was filtrated and applied to a Ni-NTA (Expedeon) column equilibrated with 1 × PBS/2 mM imidazole. The column was washed with 1 × PBS/10 mM imidazole until absorption at 280 nm reached baseline. Elution was initiated by applying PBS/250 mM imidazole. Fractions containing protein were pooled and split into aliquots that were frozen in liquid nitrogen. 0.5–1 mg of the purified proteins were thawed on ice and kept in the dark. To induce polymerization, the protein solution was reduced with 10 mM DTT and transferred into a dialysis tube (Serva, MWCO: 3.5 kD) that contained a glass coverslip. Dialysis was performed against 2 L 10 mM NaH_2_PO_4_, pH 6.9. The buffer was exchanged every 2,5 hours for 4 times. Unpolymerized proteins were removed by washing the coverslip in ddH_2_O. Protein polymers were mounted in Mowiol 4–88 (Sigma) and analyzed with a Nikon A1 confocal microscope.

For RHCC fusion proteins, *E. coli* BL21(DE3) were transformed with pET15b coding for N-CRD-RHCC-N-CRD, C-CRD-RHCC-C-CRD, or RHCC only. Induction and harvesting of cells was performed as described above. Lysis of bacteria was initiated by resuspending pellet in lysis buffer (8 M urea/50 mM Tris, pH 8.0) for 1 hour at room temperature followed by centrifugation at 22000 × g, 30 min, 4 °C. The supernatant was filtrated and applied to a Ni-NTA column equilibrated with lysis buffer. The column was washed with lysis buffer containing 10 mM imidazole. The buffer was changed gradually to PBS/10 mM imidazole in order to allow a slow refolding of the protein. When absorption at 280 nm reached baseline, elution was initiated by applying PBS/250 mM imidazole. Protein-containing fractions were supplemented with sodium azide and protease inhibitor mix (Roche) and stored at 4 °C. For reoxidation experiments, 1 mg of protein was reduced with 10 mM DTT and transferred into a dialysis tube (Serva, MWCO: 3.5 kD). As negative control, proteins were reduced and further incubated with 60 mM iodeacetamide (IAA) for 45 minutes in dark prior to dialysis. Dialysis was performed in separate beakers for each sample series as described above. Samples were taken in 30 minutes intervals for 4 hours in total. Western blotting was performed using mouse-anti-His antibody (Qiagen) at 1:2000. Blots were evaluated for polymerization kinetics using Fiji software. The background was subtracted and the intensity was measured. The intensity at the start of the incubation (t = 0) was arbitrarily set to 1 since it is considered as the maximum amount of dimer in the sample. Since the loss of dimer follows an exponential decay, the curves have been fitted using a single exponential decay model (y = e^−x/τ^).

### Immunohistochemistry

Animals were relaxed in 2% urethane in hydra medium for 2 minutes at room temperature and subsequently fixed in 4% formaldehyde/1 × PBS/0.1%Tween-20 (N-CRD and C-CRD) or Lavdovky’s fixative (N-Col 1; 50% ethanol, 10% formaldehyde, 4% acetic acid) overnight at 4 °C. Fixative was quenched by incubating animals in 50 mM NH_4_Cl/PBS for 10 minutes followed by three washes in PBS. Solubilization was initiated by incubating hydras in 1% Triton X-100 for 30 minutes. The antibodies were diluted (N-CRD and C-CRD at 1:250, NCol-1 at 1:500) in PBS/0.5% BSA/0.1% Tween-20 and incubated overnight at 4 °C. To remove unbound antibodies, three 10-minute wash steps with PBS/0.1% Tween-20 were performed. The secondary antibodies (Life Technologies) were diluted 1:400 in 1% BSA/PBS and incubated for two hours at room temperature (Alexa Fluor 488 goat anti-guinea pig for the N-CRD, Alexa Fluor 568 goat anti-rabbit for the C-CRD, Alexa Flour 488 goat anti-rabbit for NCol1). After, animals were washed three times in PBS/0.1% Tween-20 and subsequently mounted in Mowiol 4–88 (Sigma).

### Molecular Dynamics simulations

Molecular dynamics (MD) simulations were carried out on one of the N-CRD and C-CRD NMR conformers^16^,^18^ in explicit solvent upon the selective breakage of disulfide bonds, nominally, the C1-C13 and C9-C17 bridges for the N-CRD and the C1-17 and C9-C18 bridges for the C-CRD. Hydrogen atoms were added and molecular topologies were generated according to the AMBER99-sb*-ILDN force field^36^. Proteins were solvated using TIP3P water molecules and Na^+^ and Cl^−^ ions were added to achieve an ionic strength of 150 mM. The systems were then energy minimized using a steepest-descent algorithm. Following energy minimization, two equilibration runs were performed and temperature and pressure were coupled to the desired values of 300 K and 1 bar respectively. A first equilibration step was performed in the NVT ensemble with the temperature coupled at 300 K for 500 ps, using the V-rescale thermostat^37^. In this step, initial velocities were assigned to the particles composing the system. A second 500 ps-long equilibration step was performed in the NpT ensemble using the velocities previously generated. At this stage, the pressure was coupled in each dimension using a Parrinello-Rahman barostat^38^. In both NVT and NpT steps, the protein was position restrained through a restraining potential of 1000 kJ mol^−1^. 450 ns MD production runs were then performed for ten independent replicates, using a time step of 2 fs. The difference between the replicates lied in the different set of initial velocities generated at the start of the NVT equilibration step. Non-bonded interactions were computed every 10 steps from the starting of the simulations using a 1.0 nm cutoff. Coulomb interactions were computed using the particle mesh Ewald summation method. Van der Waals (vdW) interactions followed a 12-6 Lennard-Jones potential. Both interactions were computed within a 1.0 nm cutoff. All simulations were performed using GROMACS version 4.6.7^39^. Simulation results were analyzed using both in-house and GROMACS tools and molecular graphics images were created using the UCSF chimera package from the Computer Graphics Laboratory, University of California, San Francisco^40^.

### Molecular Docking

The homodimeric and heterodimeric association of the two domains was performed in both, partially reducing and fully oxidizing conditions. Starting poses were retrieved from each of the conformers available in the NMR-resolved structures. 10 conformers have been modeled for the N-CRD and the C-CRD, respectively. Each of these conformers was docked to each other using PATCHDOCK^41^,^42^ and the obtained poses were refined using FIREDOCK^43^. The blind docking of each monomer to form homodimers or heterodimers was performed under native conditions without any constraint in order to avoid bias towards any particular conformer. Poses showing RMSD lower than 0.6 nm cutoff were considered as redundant and therefore merged. The coordinates of the first 10 highest scored conformations of each run were isolated and refined with FIREDOCK through 50 Monte Carlo cycles employing a full-side chains optimization. The sub-selection of the first 10 conformations for each docking run yielded a maximum of 1000 structures per system. Out of these refined structures, the distance between all possible disulfide atoms in both monomers was computed and the docked dimers showing the lowest S-S intermolecular distance were retrieved.

### Analysis and classification of S-S bonds

The analysis of the S-S bonds in the N-CRD and C-CRD (PDB codes 1ZPX and 1SP7 for the N-CRD and C-CRD, respectively) has been performed according to the method proposed by Schmidt *et al*.^25^. In particular, the χ_1_ χ_2_ χ_3_ χ_2′_ χ_1′_ dihedral angles have been calculated for each conformer along the deposited NMR ensemble. The sign of these angles defines the class to which the bond belongs^25^. The strain energy defining bond’s reactivity has been calculated using the following empirical formula:


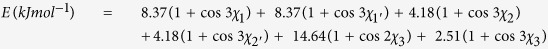


## Additional Information

**How to cite this article**: Tursch, A. *et al*. Minicollagen cysteine-rich domains encode distinct modes of polymerization to form stable nematocyst capsules. *Sci. Rep*. **6**, 25709; doi: 10.1038/srep25709 (2016).

## Supplementary Material

Supplementary Information
