# Supplementary material for: Minicollagen cysteine-rich domains encode distinct modes of polymerization to form stable nematocyst capsules
Source: Sci Rep. 2016 May 11;6:25709. doi: 10.1038/srep25709 (PMC4863159; doi:10.1038/srep25709)
Supplement: Supplementary Information [file srep25709-s1.pdf]

Supplementary Information

Minicollagen cysteine-rich domains encode distinct modes of polymerization to form stable nematocyst capsules

Anja Tursch, Davide Mercadante, Jutta Tennigkeit, Frauke Gräter and Suat Özbek

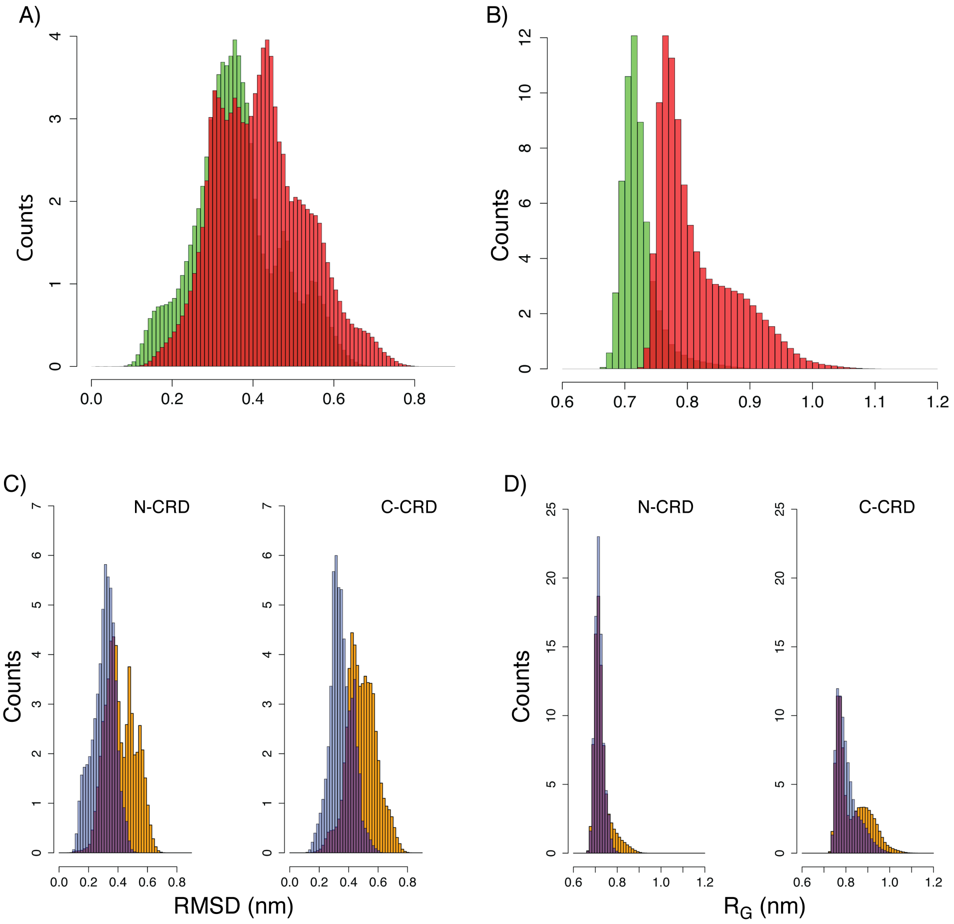

Supplementary Figure S1. Conformational dynamics of partially reduced CRDs. (A) Root mean square deviation (RMSD) and (B) radius of gyration (R_G) distributions for the N-CRD (green) and C-CRD (red) domains, as obtained from molecular dynamics simulations. The same observations are reported in panels (C) and (D) for simulated systems in which one S-S bridge has been reduced. Blue-coloured distributions indicate a reduction of the S-S bond at the N-terminus (C1-C13/C1-C17 for N-CRD and C-CRD respectively) of the domains, whereas gold-coloured distributions indicate the reduction of the S-S bond at the C-terminus of the domains (C9-C17/C9-C18 for the N-CRD and C-CRD respectively).

Supplementary Information

Minicollagen cysteine-rich domains encode distinct modes of polymerization to form stable nematocyst capsules

Anja Tursch, Davide Mercadante, Jutta Tennigkeit, Frauke Gräter and Suat Özbek

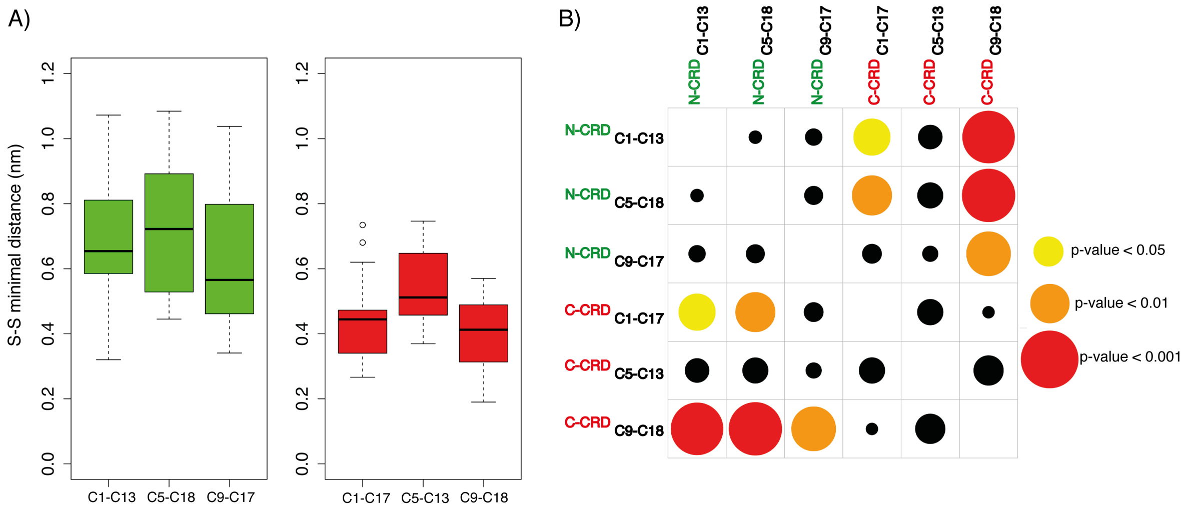

Supplementary Figure S2. Tendency of CRD disulfide bridges to engage in intermolecular disulfide bonds. (A) Intermolecular S-S minimal distances for the N-CRD (green) and C-CRD (red) disulfide bonds as retrieved from the homophilic docking of N-CRD and C-CRD domains. (B) Matrix showing the statistical significance of the comparison between the distributions shown in (A). The diameter and the colour of the circles reported in the matrix is reflective of the p-value calculated by subjecting the distributions to a two-sample Smirnov-Kolmogorov test. For clarity, the reported p-value is shown as -ln(p-value) so that the diameter of the circles increases with increasing statistically significant difference.

Supplementary Information

Minicollagen cysteine-rich domains encode distinct modes of polymerization to form stable nematocyst capsules

Anja Tursch, Davide Mercadante, Jutta Tennigkeit, Frauke Gräter and Suat Özbek

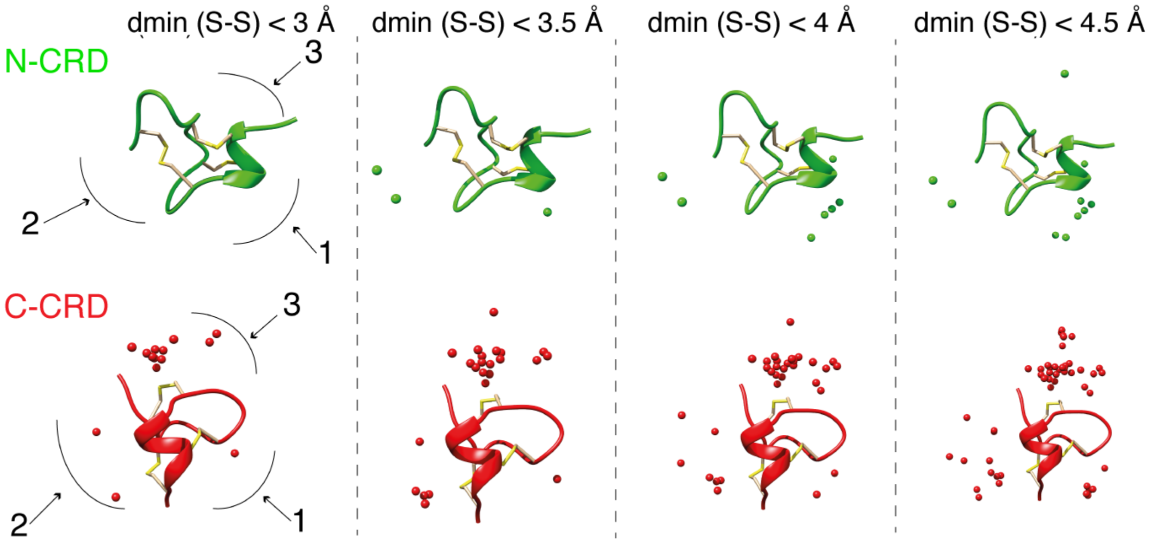

Supplementary Figure S3. Ability of C-CRD and N-CRD to function as multimerization motifs. The spheres around the structures of a N-CRD (upper panel - green) and C-CRD (lower panel - red) domains report the positions of the center of mass of the monomer docked against the one represented in cartoon. The number of docked events is filtered as a function of the minimal S-S distance encountered in the complexes. Disulfide bonds along the structures are represented as balls and sticks and coloured by atom types: carbon atoms are coloured in beige and sulphur atoms are coloured in yellow.

Supplementary Information

Minicollagen cysteine-rich domains encode distinct modes of polymerization to form stable nematocyst capsules

Anja Tursch, Davide Mercadante, Jutta Tennigkeit, Frauke Gräter and Suat Özbek

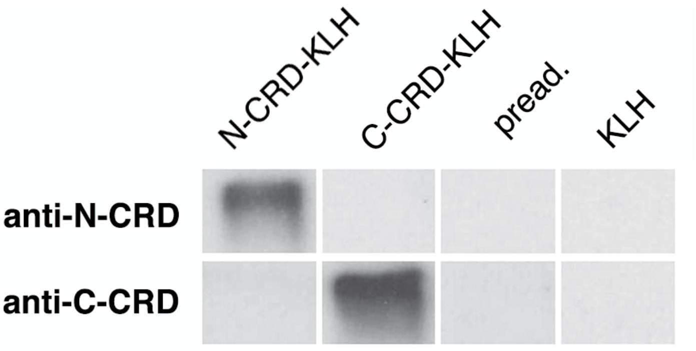

Supplementary Figure S4. Specificity of affinity-purified CRD antibodies. Key-Hole-Limped (KLH)-conjugated N-CRD and C-CRD peptides were analyzed by Western blot using specific N-CRD and C-CRD antibodies. Unconjugated KLH was applied as control. Pre-adsorption (preads.) of the antibodies with the corresponding antigenic peptides abolished immunoreactivity for the CRD domains.

Supplementary Information

Minicollagen cysteine-rich domains encode distinct modes of polymerization to form stable nematocyst capsules

Anja Tursch, Davide Mercadante, Jutta Tennigkeit, Frauke Gräter and Suat Özbek

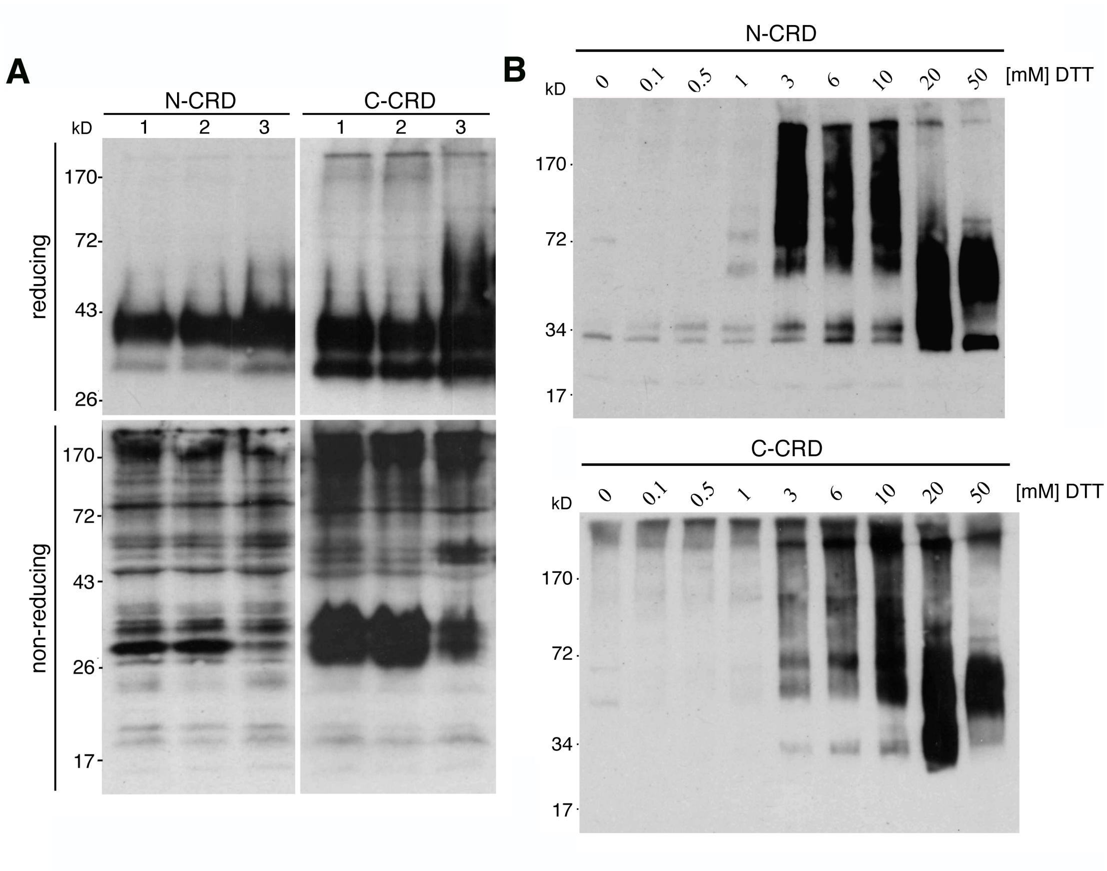

Supplementary Figure S5. Partial reduction of minicollagen-1 polymers in isolated nematocysts monitored by N- and C-CRD antibodies. A, detection of NCol-1 with CRD antibodies in different tissue lysates under reducing and non-reducing conditions. C-CRD antibody showed higher reactivity for NCol-1 in all samples. Monomeric NCol-1 protein exhibits an apparent molecular mass of about 30kDa in SDS-PAGE due to a retarded mobility of collagens. The predicted molecular mass of mature NCol-1 is 14 kDa. The pronounced double band is caused by the difference in gel mobility of the reduced and oxidized CRDs. 1, whole hydra; 2, gastric region; 3, isolated nematocysts. B, isolated nematocysts were incubated with increasing concentrations of DTT as indicated. Released minicollagen-1 oligomers were detected using either N- or C-CRD antibody. Antibody accessible CRDs were of higher abundance in the N-CRD Western blot.

Supplementary Information

Minicollagen cysteine-rich domains encode distinct modes of polymerization to form stable nematocyst capsules

Anja Tursch, Davide Mercadante, Jutta Tennigkeit, Frauke Gräter and Suat Özbek

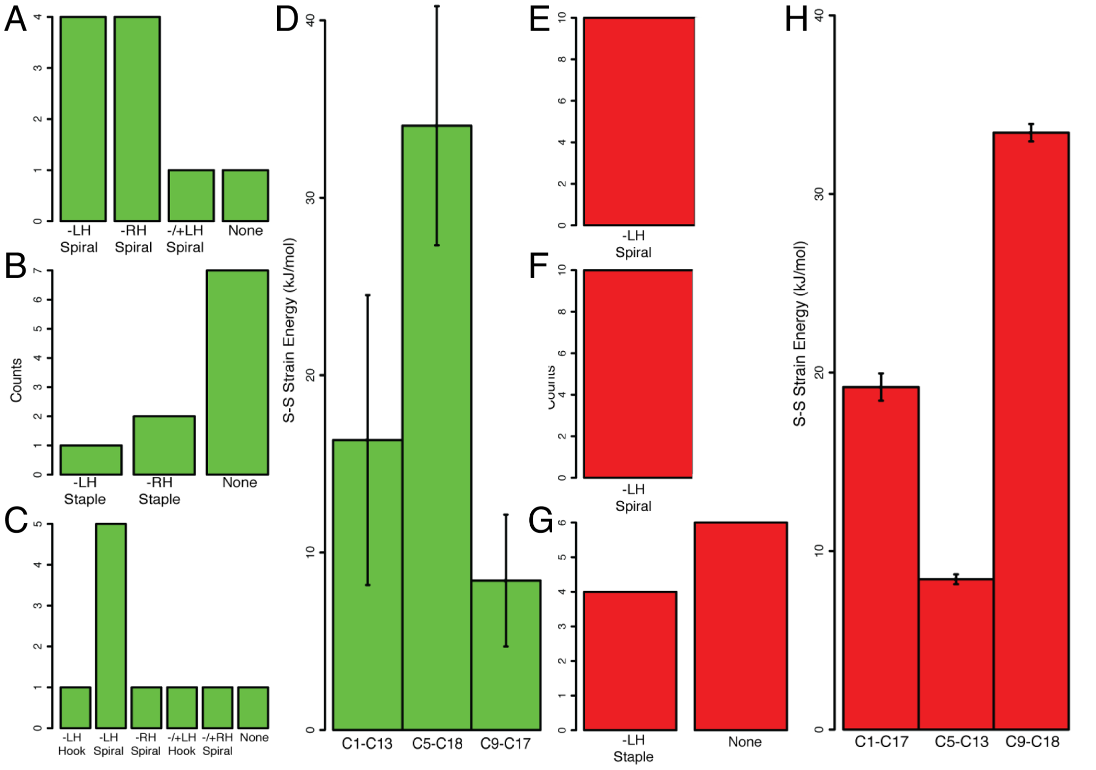

Supplementary Figure S6. Analysis of S-S bonds along N-CRD and C-CRD NMR conformers. The disulfide bond classification is reported for the C1-C13, C5-C18 and C9-C17 S-S bonds of the N-CRD (1ZPX) (panels A-C) and for the C1-C17, C5-C13 and C9-C18 of the C-CRD domain (1SP7) (panels E-F). The class to which the disulfide bonds belong (see Methods for details) in a particular conformation of the domain is reported below the bars. The bar heights represent the conformer counts along the ensemble that shows a disulfide bond falling within a particular class. "None" refers to the in-ability to classify the bond in any of the previously described classes by Schmidt *at al.* {Schmidt, 2006 #1218}. According to the values of the χ1 to χ5 angles the strain energy of each bond in each conformer has been calculated and the average and standard deviations across the ensemble are reported in panels D and H.

Supplementary Information

Minicollagen cysteine-rich domains encode distinct modes of polymerization to form stable nematocyst capsules

Anja Tursch, Davide Mercadante, Jutta Tennigkeit, Frauke Gräter and Suat Özbek

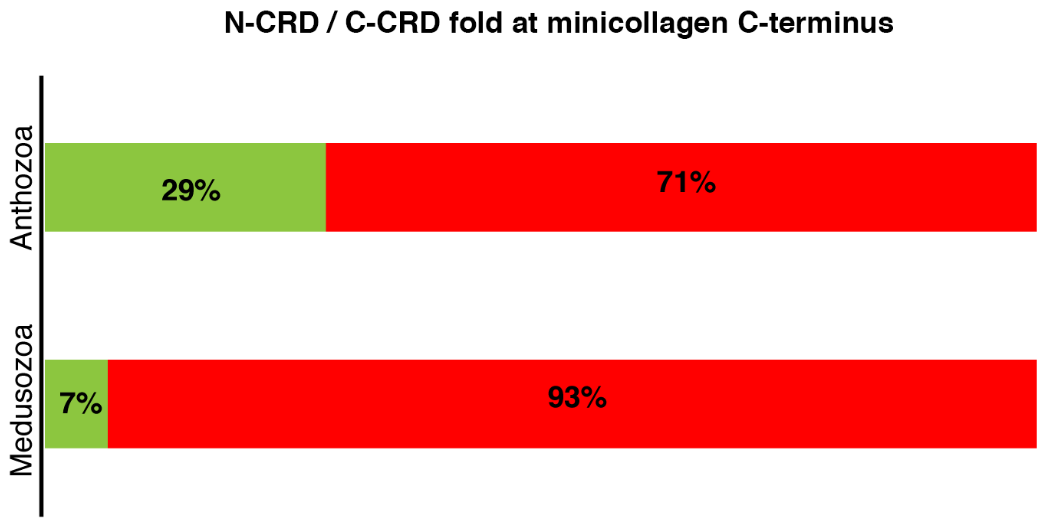

Supplementary Figure S7. C-CRD fold is prevalent in the medusozoan lineage. C-terminal CRDs of available minicollagen sequences (N=14 for anthozoa; N=18 for medusozoa) were examined for C-CRD (red bar) or N-CRD identity (green bar) and expressed as relative frequency. While almost one third of the anthozoan C-terminal minicollagen CRDs possess the N-CRD fold, the medusozoan clade predominantly exhibits the C-CRD fold.

Supplementary Information

Minicollagen cysteine-rich domains encode distinct modes of polymerization to form stable nematocyst capsules

Anja Tursch, Davide Mercadante, Jutta Tennigkeit, Frauke Gräter and Suat Özbek

Supplementary Materials and Methods

Nematocyst Isolation

Hydras were collected in a 1.5ml tube, the medium was removed and animals were frozen overnight at -80°C. On the next day, hydras were homogenized with a syringe in homogenization buffer (50% Percoll, 10% sucrose, 0.003% Triton-X 100) and subsequently spun down at 7500 rpm for 15 minutes at 4°C. The pellet was again homogenized and centrifuged as described before. Thereafter, the pellet was washed once in washing buffer (1xPBS, 0.003% Triton-X 100, 10% sucrose) and centrifuged at 7500 rpm for 10 minutes at room temperature. The resulting pellet was resuspended in 1xPBS/ 10% Sucrose. The capsule concentration was determined by using a Neubauer counting chamber.

Western Blotting

For testing antibody specificity 2ng of KLH-conjugated CRD peptides (N-CRD: ANPCGSYCPSVCAPACAPVCCY, C-CRD: CPPVCVAQCVPTCP QYCCPA)

and non-conjugated KLH protein were mixed with reducing sample buffer, heated at 95°C for 10 minutes and subsequently separated on a 10% SDS gel. Proteins were transferred onto a nitrocellulose membrane (Amersham Protran 0.45 NC, GE Healthcare) at 360 mA for 1,5 hours. Complete transfer of proteins was confirmed by Poinceau S stain. Membranes were blocked with blocking buffer (5% BSA, 0.8x PBS, 0.1% Tween-20) for one hour at RT. Antibodies specific for either N-CRD or C-CRD were applied 1:1000 to 10ml blocking buffer and incubated overnight at 4°C. Membranes were washed three times with 1xPBS/ 0.1% Tween-20 for ten minutes. Secondary antibody (donkey-anti-guinea pig for N-CRD; goat-anti-rabbit in terms of C-CRD, *Jackson Immuno Research*) was applied at 1:5000 in blocking solution and incubated for 1 hour at RT. Membranes were washed twice with 1x PBS/0.1% Tween-20 and once with 1xPBS for ten minutes each. ECL1 (100mM Tris, pH 8.0, 5mM H_2O_2) and ECL2 (125mM Tris pH 8.0, 2mM luminol, 400µm coumaric acid) were mixed 1:1 and applied on the membrane. Chemiluminescence signals were detected by exposing light-sensitive films (Amersham Hyperfilm ECL, GE Healthcare) to membranes. The same protocol was followed for antibodies that were preadsorbed with their respective antigen for 6 hours at 4°C prior to incubation with blotted membrane.
